# Supplementary material for: Astrovirus in Reunion Free-Tailed Bat (Mormopterus francoismoutoui)
Source: Viruses. 2021 Aug 2;13(8):1524. doi: 10.3390/v13081524 (PMC8402754; doi:10.3390/v13081524)
Supplement: Supplementary file 1 [file viruses-13-01524-s001.zip › viruses-1268731-supplementary.pdf]

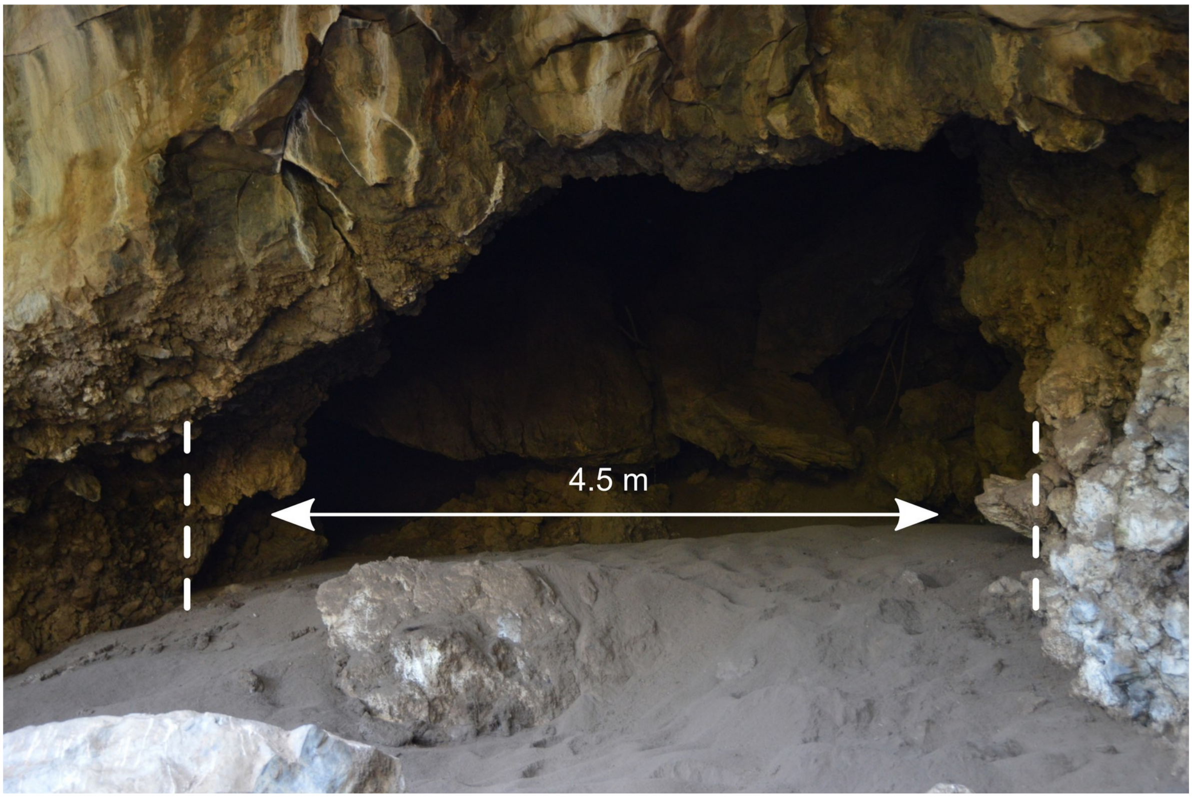


**Figure S1.** Cave entrance of the studied colony. The narrowest part of the entrance is approximately 4.5 m width and 2m high.

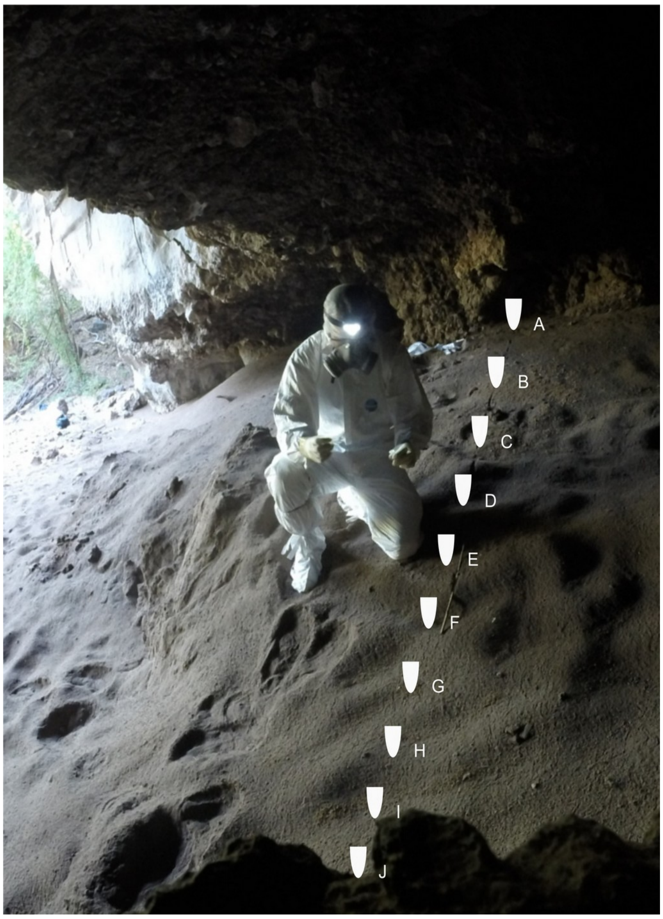


**Figure S2.** Sampling transect at the cave entrance. Ten guano samples separated by 50 cm (A to J) were collected in each sampling session.
